# Population affinity estimation using pelvic measurements based on computed tomographic data acquired from Japanese and Western Australian populations

**DOI:** 10.1007/s00414-024-03178-3

**Published:** 2024-02-06

**Authors:** Suguru Torimitsu, Akari Nakazawa, Ambika Flavel, Lauren Swift, Yohsuke Makino, Hirotaro Iwase, Daniel Franklin

**Affiliations:** 1 Centre for Forensic Anthropology, University of Western Australia, Crawley, WA 6009 Australia; 2 Department of Forensic Medicine, Graduate School of Medicine, The University of Tokyo, Tokyo, 113-0033 Japan; 3 Department of Obstetrics and Gynecology, Graduate School of Medicine, The University of Tokyo, Tokyo, 113-8655 Japan

**Keywords:** Population affinity estimation, Pelvis, Computed tomography, Japanese, Western Australia

## Abstract

The present study analyzes morphological differences in the pelvis of Japanese and Western Australian individuals and investigates the feasibility of population affinity classification based on computed tomography (CT) data. The Japanese and Western Australian samples comprise CT scans of 207 (103 females; 104 males) and 158 (78 females; 80 males) adult individuals, respectively. Following volumetric reconstruction, a total of 19 pelvic landmarks were obtained on each sample, and 11 measurements, including two angles, were calculated. Machine learning methods (random forest modeling [RFM] and support vector machine [SVM]) were used to classify population affinity. Classification accuracy of the two-way models was approximately 80% for RFM: the two-way sex-specific and sex-mixed models for SVM achieved > 90% and > 85%, respectively. The sex-specific models had higher accurate classification rates than the sex-mixed models, except for the Japanese male sample. The classification accuracy of the four-way sex and population affinity model had an overall classification accuracy of 76.71% for RFM and 87.67% for SVM. All the correct classification rates were higher in the Japanese relative to the Western Australian sample. Our data suggest that pelvic morphology is sufficiently distinct between Japanese and Western Australian individuals to facilitate the accurate classification of population affinity based on measurements acquired in CT images. To the best of our knowledge, this is the first study investigating the feasibility of population affinity estimation based on CT images of the pelvis, which appears as a viable supplement to traditional approaches based on cranio-facial morphology.

## Introduction

Determining the identity of unidentified remains is crucial in forensic investigations, especially while analyzing severely mutilated and skeletal remains [1]. Population affinity estimation is one of the four basic steps of creating a biological profile and determining sex, age, and stature [2]. Although estimating population affinity is challenging [3], it can narrow down the list of missing persons by comparing skeletal remains with dental records, medical records, and other data [4].

Previous research has documented skeletal morphological differences between and within individuals from global populations. In terms of ancestral variances specifically, the skeletal region of primary consideration is the skull, especially the midface [5–11]. The pelvis, however, also has unique heterogeneous characteristics and is one of the most diagnostic bones for anthropological profiling, particularly in the estimation of age and sex [12–18]. The forensic utility of the pelvis is also inherently related to postmortem survivability associated with large musculature that affords some degree of protection to the os coxae and proximal femora; accordingly, morphometric data from those bones can commonly be collected by forensic anthropologists [19].

Pelvic differences can be assessed by qualitative (morphoscopic) and/or quantitative (morphometric) methods [20–22]. The former involves the visual analysis of structural differences, but can be limited by definitions that are often ill-defined and/or have unclear scoring procedures [23]. Additionally, morphoscopic methods are subjective and require appropriate observer experience [21]. Conversely, although quantitative morphometric analysis of the pelvis tends to be more time-consuming and complex than qualitative methods, it is less subjective and is generally associated with lower inter- and intra-observer error [20, 21]. Interestingly, however, there appears a paucity of research that has specifically evaluated morphometric differences in the pelvis between individuals from different populations [13–15]. Although Asia comprises individuals from many countries and of different ethnicities presenting obvious phenotypic variability in body size and shape [24], there have been no reports comparing the pelvic measurements between Asians, including Japanese, and other populations.

Computed tomography (CT) can capture high-level details of bony structures without the need to remove the soft tissue, thus offering a time-saving alternative to a physical forensic examination and concurrently protecting remains from further invasive manipulation [24, 25]. Data obtained using CT images are more representative of the contemporary population compared to documented skeletal collections based on body donation programs. This is largely because digital data from modern individuals is less likely to be influenced by secular population variances [26] compared to historical skeletal collections. Moreover, multiple studies have demonstrated that the use of CT images provides appropriate levels of reproducibility and accuracy, and that medical imaging offers an appropriate proxy to physical remains for the formulation of forensic standards designed to facilitate biological profiling of unknown skeletal remains [27].

The present study quantifies morphological differences between pelves of contemporary Japanese and Western Australian populations and thereafter evaluates the feasibility of population affinity classification based on morphometric data obtained from multidetector CT (MDCT) images using machine learning statistical approaches.

## Materials and methods

### Materials

#### Japanese population

The sample comprises postmortem CT (PMCT) scans of 207 adult corpses over 18 years of known age and sex (103 females, mean age = 40.76 ± 11.55 years; 104 males, mean age = 38.46 ± 8.68 years) from the Department of Forensic Medicine at the University of Tokyo between August 2017 and May 2022. The estimated postmortem interval for all the subjects was < 14 days. The exclusion criteria were fractures of the pelvis, a pubic symphysis diastasis, sacroiliac joint dislocations, burn injuries, bone implant, and acquired or congenital abnormalities. The study protocol was approved by the Ethics Committee of the University of Tokyo (2121264NI).

#### Western Australian population

The sample comprises MDCT scans of 158 adult individuals (78 females, mean age = 45.05 ± 18.17 years; 80 males, mean age = 41.23 ± 20.46 years) who presented at one of the major Western Australian hospitals (Perth region) for clinical evaluation between April 2009 and July 2012. In accordance with the National Statement on Ethical Conduct in Human Research (National Statement), the scans were anonymized, retaining only sex and age information. Although specific information on the ethnicity of each individual was not maintained in the patient data, the entire sample was taken as representative of a “typical” Western Australian population [28]. Exclusion criteria are the same as stated for the Japanese population. Research ethics approval was granted by the Human Research Ethics Committee of the University of Western Australia (2020/ET000038).

### Methods

For the Japanese subjects, PMCT scanning was performed with a 16-row detector CT system (Eclos; Fujifilm Healthcare Corporation, Tokyo, Japan). The scanning protocol was as follows: collimation of 1.25 mm, reconstruction interval of 1.25 mm, tube voltage of 120 kV, and tube current of 200 mA. For Western Australian subjects, pelvic imaging was performed using a 64-slice CT scanner (Brilliance; Phillips Healthcare, NSW, Australia) with an average slice thickness of 0.94 mm, tube voltage of 100–140 kV, and automatic tube current modulation. The images were reconstructed with uniform thickness (an average thickness of 0.94 mm).

**Fig. 1:**
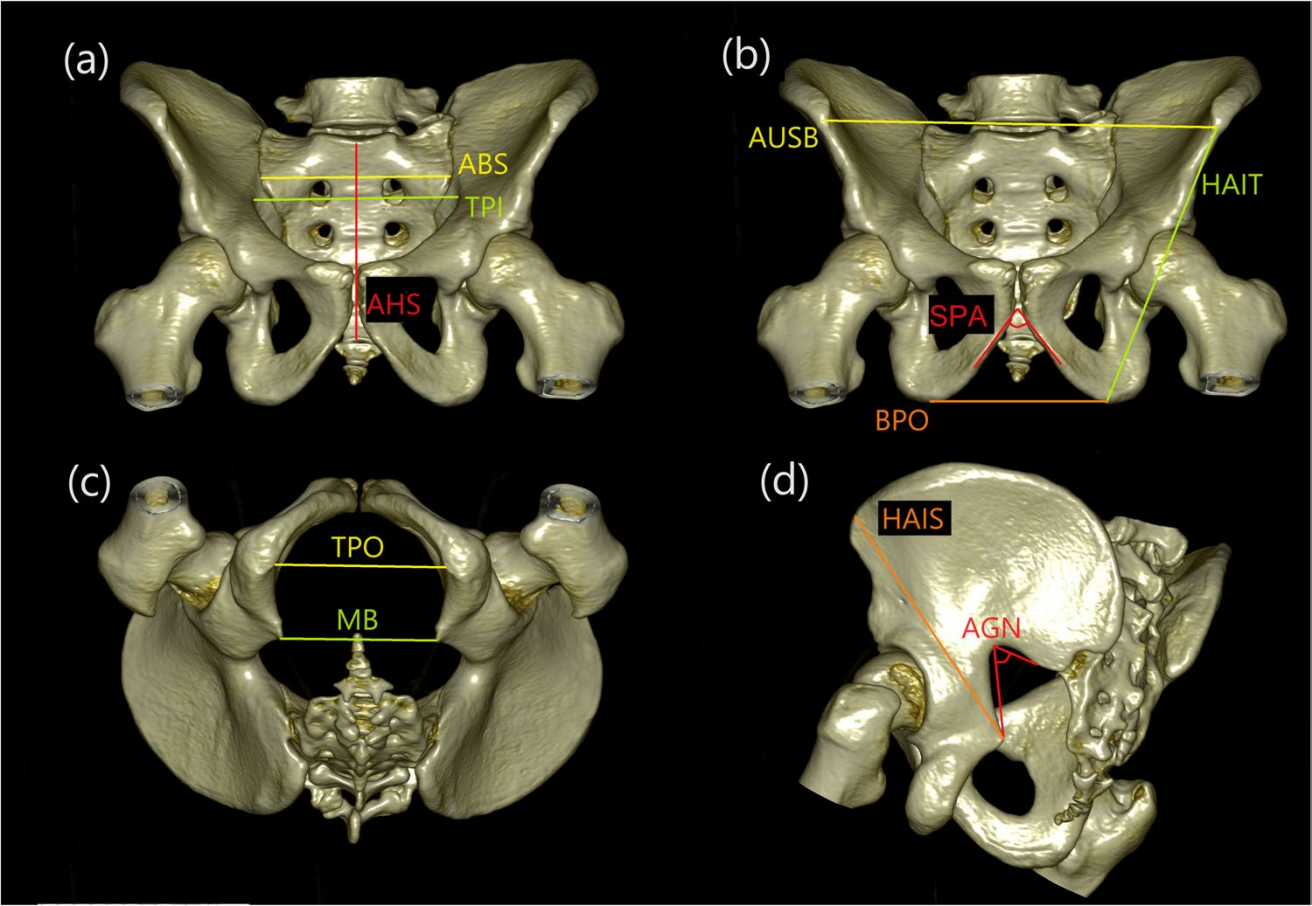
Three-dimensional computed tomography images showing pelvic measurements (see Table 2 for definition): **a** transverse pelvic inlet (TPI), anterior breadth of the sacrum (ABS), and anterior height of the sacrum (AHS); **b** subpubic angle (SPA), breadth of pelvic outlet (BPO), anterior upper spinal breadth of pelvis (AUSB), and height between anterior superior iliac spine and anteroinferior margin of ischial tuberosity (HAIT); **c** transverse pelvic outlet (TPO) and midpelvic breadth (MB); and **d** angle of greater sciatic notch (AGN) and height between anterior superior iliac spine and ischial spine (HAIS)

Image data processing and three-dimensional (3D) volume rendering were performed on a workstation (OsiriX MD version 11.0.2; Pixmeo SARL, Geneva, Switzerland). Soft tissue kernel was used for the acquisition of the CT. In accordance with previous studies [25, 29, 30], 19 pelvic landmarks (Table 1) were obtained on each sample. Using MorphDB (an in-house developed database application) and Excel software (Microsoft Office 2019, Microsoft, Redmond, WA, USA), 11 measurements, including two angles, were calculated using coordinates of the landmarks obtained in 3D images (Table 2 and Fig. 1).

**Table 1:** Definitions of the landmarks of the pelvis

Landmark (abbreviation)	Definition
Left pelvic inlet (lpi)	The most lateral (left) point on the interior of the pelvic brim (superior–inferior aspect) [24]
Right pelvic inlet (rpi)	The most lateral (right) point on the interior of the pelvic brim (superior–inferior aspect) [24]
Left pelvic outlet (lpo)	The medial inferior point of the left ischial tuberosity corresponding to the maximum width of the pelvic outlet (inferior-superior aspect) [24]
Right pelvic outlet (rpo)	The medial inferior point of the right ischial tuberosity corresponding to the maximum width of the pelvic outlet (inferior–superior aspect) [24]
Left anterior sacrum (lsa)	The most lateral (left) and anterior point of the sacrum at the level of the auricular surface [28]
Right anterior sacrum (rsa)	The most lateral (right) and anterior point of the sacrum at the level of the auricular surface [28]
Sacral promontory (sp)	The most superior and anterior point of the sacrum in the median sagittal plane [24]
Sacral-coccyx border (b)	The most anterior point of the sacral-coccyx border [29]
Pubic symphysis (p)	The most inferior anterior point on the symphyseal surface [28]
Left ischiopubic ramus (lir)	The most inferior point on the left ischiopubic ramus [28]
Right ischiopubic ramus (rir)	The most inferior point on the right ischiopubic ramus [28]
Posterior inferior iliac spine (pi)	The left point on the most inferior spine on the ilium where the smooth arc of the greater sciatic notch ends medially [28]
Deepest point (d)	The deepest point in the left greater sciatic notch [24]
Left ischial spine (lis)	The tip on the left ischial spine [28]
Right ischial spine (ris)	The tip on the right ischial spine [28]
Left ischial tuberosity (lt)	The most inferior point on the left ischial tuberosity in the median sagittal plane [28]
Right ischial tuberosity (rt)	The most inferior point on the right ischial tuberosity in the median sagittal plane [28]
Left anterior superior iliac spine (la)	The point at the left anterior superior iliac spine [28]
Right anterior superior iliac spine (ra)	The point at the right anterior superior iliac spine [28]

**Table 2:** Definitions of the pelvic measurements (see Table 1 for landmark definition)

Measurement	Definition	Landmarks
Transverse pelvic inlet (TPI)	The widest transverse distance of the pelvic inlet [24]	lpi-rpi
Transverse pelvic outlet (TPO)	The widest transverse distance of the pelvic outlet [24]	lpo-rpo
Anterior breadth of the sacrum (ABS)	Maximum transverse distance of the sacrum at the anterior projection of the auricular surface [24]	lsa-rsa
Anterior height of the sacrum (AHS)	Distance between the midpoint of the sacral promontory and sacral/coccyx border [24]	sp-b
Subpubic angle (SPA)	Angle formed by the inferior pubic symphysis and the ischiopubic ramus on bilateral sides [29]	lir-p-rir
Angle of greater sciatic notch (AGN)	Angle formed by the left posterior inferior iliac spine, the deepest portion of the greater sciatic notch, and the ischial spine [28]	pi-d-lis
Midpelvic breadth (MB)	The linear distance between the tips of the left and right ischial spines [28]	lis-ris
Breadth of pelvic outlet (BPO)	The linear distance between the inferior margins of the left and right ischial tuberosities [28]	lt-rt
Anterior upper spinal breadth of pelvis (AUSB)	The linear distance between the left and right anterior superior iliac spines [28]	la-ra
Height between anterior superior iliac spine and ischial spine (HAIS)	The linear distance from the left anterior superior iliac spine to the tip of the left ischial spine [28]	la-lis
Height between anterior superior iliac spine and anteroinferior margin of ischial tuberosity (HAIT)	The linear distance from the left anterior superior iliac spine to the anteroinferior margin of the left ischial tuberosity [28]	la-lt

A subset of six subjects (three females and three males) were randomly selected to assess intra- (ST) and inter-observer errors (AN). All 19 pelvic landmarks were obtained on each of the six subjects, and this process was repeated six times, with a minimum of two-day intervals. The acquisition order of landmarks was changed each time to reduce recall between repetitions. Subsequently, the relative technical error of measurement (rTEM, %) and coefficient of reliability (*R*) were calculated. The acceptable rTEM range outlined by established anthropological studies [31–33] was taken to be < 5%; *R* values > 0.75 were considered sufficiently precise [34, 35].

Descriptive statistics, including ranges, mean, standard deviation, and median for each set of measurements, for both sexes, were calculated. The Brunner–Munzel test was used to determine if significant differences existed between the two groups; a *p* value of < 0.05 was considered statistically significant. Two machine learning methods were employed for population affinity classification: (i) random forest modeling (RFM), which belongs to a class of machine learning techniques comprising traditional classification trees created using nonparametric algorithms that incorporate majority voting and bagging to assign cases to response classes [36–38], and (ii) support vector machine (SVM), which uses data located at the edge of the multivariate space (the intersection of two groups) to generate classification rules by maximizing the margin between the two groups [39, 40].

The utility of machine learning models was examined in the following two scenarios: (i) two-way models distinguished by sex-specific and sex-mixed population affinity and (ii) a four-way model distinguished by population affinity and sex simultaneously. Regarding RFM, the random forest feature importance during the analysis was also calculated. All machine learning performances were analyzed using R 4.2.3 (R Foundation for Statistical Computing, Vienna, Austria) with the “randomForest” and “e1071” packages [41, 42].

## Results

The rTEM and the *R* values were 0.19%–1.81% and 0.980–0.999, respectively (Table 3). The mean, range, and standard deviation values of the 11 measurements for both sexes are presented in Table 4 and 5. In considering the female sample, it was evident that four measurements (transverse pelvic inlet (TPI), anterior breadth of the sacrum (ABS), height between anterior superior iliac spine and ischial spine (HAIS), and height between anterior superior iliac spine and anteroinferior margin of ischial tuberosity (HAIT)) were significantly smaller in the Japanese population. The angle of the greater sciatic notch (AGN) and breadth of the pelvic outlet (BPO) were significantly larger compared to the Western Australian individuals (Table 4). For the Japanese male sample, four measurements (ABS, AGN, HAIS, and HAIT) were significantly smaller. Conversely, transverse pelvic outlet (TPO), midpelvic breadth (MB) and BPO were significantly larger than the corresponding data for the Western Australian male individuals (Table 5). No significant population affinity differences were observed in other measurements.

**Table 3:** Relative technical error of measurements (rTEM) and coefficient of reliability (*R*)

Measurement	Intra-observer error		Inter-observer error	
	rTEM	*R*	rTEM	*R*
TPI	0.20%	0.999	0.23%	0.999
TPO	0.25%	0.999	0.38%	0.999
ABS	0.37%	0.995	0.40%	0.994
AHS	0.19%	0.997	0.35%	0.990
SPA	1.77%	0.990	1.61%	0.992
AGN	1.81%	0.981	1.80%	0.980
MB	0.43%	0.999	0.43%	0.999
BPO	0.64%	0.997	0.69%	0.996
AUSB	0.20%	0.999	0.26%	0.998
HAIS	0.22%	0.995	0.22%	0.995
HAIT	0.30%	0.995	0.25%	0.997

**Table 4:** Descriptive statistics of 11 pelvic measurements for the female sample

	Japanese (*n* = 103)			Western Australian (*n* = 78)		
Measurement	Range	Mean ± SD^a^	Median	Range	Mean ± SD	Median
TPI (mm)	108.95–143.80	125.45 ± 7.28	125.68^b^	112.60–158.58	128.67 ± 9.10	127.89
TPO (mm)	108.77–149.15	129.57 ± 9.88	129.33	103.88–148.28	127.13 ± 10.37	127.60
ABS (mm)	85.59–121.10	107.10 ± 6.64	107.18^b^	93.63–140.33	111.06 ± 7.90	110.99
AHS (mm)	81.89–144.35	110.63 ± 10.77	110.31	82.50–137.05	111.62 ± 12.18	110.51
SPA (°)	80.92–140.80	114.19 ± 10.01	114.42	93.34–140.19	115.22 ± 10.80	114.65
AGN (°)	75.48–109.11	93.15 ± 7.17	93.09^c^	69.50–108.78	87.14 ± 8.23	86.47
MB (mm)	86.31–126.26	108.70 ± 8.04	108.54	92.01–127.81	108.27 ± 8.36	107.94
BPO (mm)	92.56–139.35	112.13 ± 9.59	112.50^c^	77.47–123.32	101.76 ± 12.11	103.68
AUSB (mm)	179.66–280.04	232.30 ± 17.37	234.76	185.51–298.88	228.97 ± 18.79	229.58
HAIS (mm)	121.96–145.32	134.78 ± 5.50	134.72^b^	120.70–153.75	139.77 ± 7.82	141.35
HAIT (mm)	143.56–178.21	162.69 ± 7.45	164.01^b^	145.62–184.37	168.06 ± 9.16	169.18

^a^Standard deviation

^b^Significantly smaller than that of Western Australian (*p* < 0.05 using Brunner–Munzel test)

^c^Significantly larger than that of Western Australian (*p* < 0.05 using Brunner–Munzel test)

**Table 5:** Descriptive statistics of 11 pelvic measurements for the male sample

	Japanese (*n* = 104)			Western Australian (*n* = 80)		
Measurement	Range	Mean ± SD^a^	Median	Range	Mean ± SD	Median
TPI (mm)	106.26–139.20	119.36 ± 6.06	118.84	103.21–144.33	121.13 ± 8.28	121.17
TPO (mm)	94.44–134.92	111.01 ± 8.36	110.30^b^	81.55–123.52	101.79 ± 10.09	101.63
ABS (mm)	91.06–122.83	107.53 ± 5.40	107.05^c^	90.60–127.98	109.50 ± 7.84	109.48
AHS (mm)	94.56–145.45	119.31 ± 10.35	119.01	90.88–146.81	116.90 ± 10.75	116.91
SPA (°)	60.03–103.18	83.24 ± 9.31	83.13	57.43–107.62	80.87 ± 10.94	80.35
AGN (°)	49.92–93.26	71.18 ± 7.20	71.64^c^	52.46–102.07	75.90 ± 9.50	75.61
MB (mm)	77.90–114.25	93.64 ± 7.50	92.65^b^	70.87–112.04	90.72 ± 7.82	91.11
BPO (mm)	74.47–132.85	94.22 ± 9.80	94.02^b^	69.45–110.56	87.00 ± 9.20	87.18
AUSB (mm)	177.96–266.84	230.96 ± 18.05	231.38	188.51–271.97	230.79 ± 19.80	231.74
HAIS (mm)	132.78–161.75	145.77 ± 6.45	145.92^c^	134.75–167.82	151.95 ± 7.41	152.09
HAIT (mm)	165.06–193.08	178.41 ± 6.39	178.57^c^	161.25–208.32	184.83 ± 9.83	184.05

^a^Standard deviation

^b^Significantly larger than that of Western Australian (*p* < 0.05 using Brunner–Munzel test)

^c^Significantly smaller than that of Western Australian (*p* < 0.05 using Brunner–Munzel test)

The results of the machine learning models are presented in Table 6 and 7. The accuracy of the two-way models was approximately 80% for RFM, and for the two-way sex-specific and sex-mixed models for SVM it was > 90% and > 85%, respectively. The sex-specific models had higher correct classification rates than the sex-mixed models, except for the Japanese male sample. The four-way model demonstrated an overall classification accuracy of 76.71% for RFM and 87.67% for SVM. All the correct classification rates were higher in the Japanese relative to the Western Australian sample.

**Table 6:** Classification matrix showing the classification of groups according to population affinity (two-way models)

		RFM			SVM		
Sex	Group	JP	WA	% Correct	JP	WA	% Correct
Female	JP	84	19	81.55%	100	3	97.09%
	WA	22	56	71.79%	14	64	82.05%
	All			77.35%			90.61%
Male	JP	90	14	86.54%	103	1	99.04%
	WA	21	59	73.75%	17	63	78.75%
	All			80.98%			90.22%
Total	JP	175	32	84.54%	192	15	92.75%
	WA	49	109	68.99%	39	119	75.32%
	All			77.81%			85.21%

*RFM* random forest modeling, *SVM* support vector machine, *JP* Japanese, *WA* Western Australian

**Table 7:** Classification matrix showing the classification of groups according to population affinity and sex (four-way models)

	RFM					SVM				
Group	JPF	JPM	WAF	WAM	% Correct	JPF	JPM	WAF	WAM	% Correct
JPF	82	2	19	0	79.61%	101	0	2	0	98.06%
JPM	1	86	3	14	82.69%	1	97	0	6	93.27%
WAF	19	4	55	0	70.51%	18	0	59	1	75.64%
WAM	0	22	1	57	71.25%	0	17	0	63	78.75%
All					76.71%					87.67%

*JPF* Japanese female, *JPM* Japanese male, *WAF* Western Australian female, *WAM* Western Australian male

**Fig. 2:**
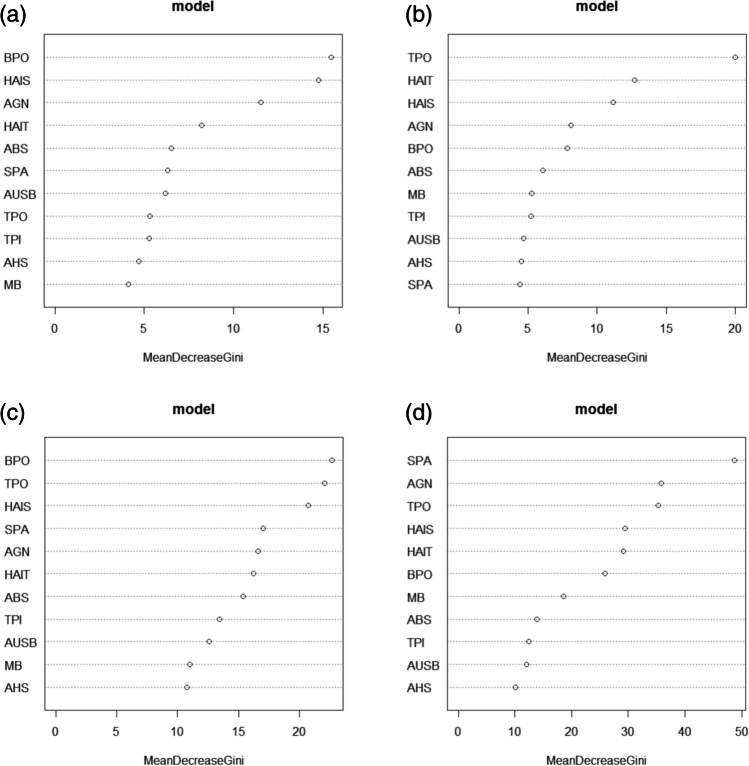
Random forest feature importance (mean decrease Gini) for the response variable: **a** the two-way female model, **b** the two-way male model, **c** the two-way sex-mixed model, and **d** the four-way sex and population affinity model

The random forest feature importance demonstrated that BPO and TPO were the strongest weighted measurements for correct classifications (express the greatest population variance) in the female and male samples, respectively (Fig. 2). Contrarily, the subpubic angle (SPA) had the highest mean decrease Gini in the four-way model, followed by AGN.

## Discussion

In this study, the intra- and inter-observer errors were small and can be considered negligible, thereby indicating that pelvic landmark and measurement acquisition using 3D CT images is precise and reproducible. Significant measurement variances between the two populations were identified in various measurement values. The superior aspect of the pelvic cavity and the vertical direction of the pelvis were larger in the Western Australian population, whereas the inferior aspect of the pelvic cavity was larger in the Japanese sample. Patriquin et al. [13] reported that the pelves of South African whites were generally larger than those of South African blacks. Furthermore, Handa et al. [14] reported that white females had a wider pelvic inlet and outlet, and shallower antero-posterior diameter than African-American females. It is evident therefore that those results suggest potential for considerable differences in pelvic measurement values among different populations.

The present study demonstrated that the classification accuracy achieved for assigning the Japanese and Western Australian individuals to their respective population of origin was > 75% and > 85% based on RFM and SVM, respectively. Therefore, although phenotypic population differences are known to be most evident in the skull [13], pelvic measurements hold obvious forensic utility for classifying Japanese and Western Australian individuals when the complete skull is absent. Further, correct classification rates were higher for Japanese individuals compared to the Western Australians. Franklin and Flavel [43] noted that from Southeast Asia. Consequently, the correct classification rates for Western Australia would likely be expected to be lower due to the heterogeneity of that population. Conversely, classification accuracy for Japanese individuals is higher, thus suggesting that the Japanese population is relatively less mixed.

Patriquin et al. [13] distinguished black and white South African left os coxae using discriminant function analysis and achieved 85% and 88% average accuracies for females and males, respectively. İşcan [12] reported classification accuracy rates of 83% in males and 88% in females using three pelvic measurements in an African American and Caucasian American population. However, TPO/BPO, which both were found to be highly diagnostic of population affinity in the two-way models of the present study, were not included in previous research. Further research is required because TPO/BPO may be useful in other populations as well. Torimitsu et al. [29] demonstrated that MB can be used to classify sex with an associated classification accuracy of > 80% in a Japanese population. However, the results of this study confirmed that MB was a less important measurement for population affinity estimation. Contrarily, SPA and AGN were the most important variables in the four-way model to distinguish population affinity and sex simultaneously. Similarly, Small et al. [15] quantified the SPA in a South African population and demonstrated statistically significant differences not only between males and females, but also between blacks and whites. These results may be mainly due to the sexual dimorphism of the SPA [24, 25, 44–46]. According to Torimitsu et al. [29], SPA and AGN contributed most significantly to sex classification in a Japanese population (accuracy rate of 98.1%). Additionally, Franklin et al. [30] reported that the SPA and AGN could classify sex with 93.2% and 85.2% cross-validated accuracy rates in Western Australian individuals, respectively. However, when applying the discriminant formula using the SPA and AGN for Japanese individuals to those from Western Australia, the sex classification accuracy was as low as 53.5% [43].

In considering the latter results, the pelvis may differ considerably morphologically depending on population of origin, albeit it is still an important element for the estimation of sex, but seemingly based on increasing empirical evidence, population affinity as well. However, relative to the estimation of population affinity based on morphometric data, it should be noted that some populations are not well described in the literature. Regardless, it is essential to consider that population differences exist in pelvic morphology due to genetics, nutritional status and environmental factors, including climates [47–55]. Thus, a more comprehensive database, including diverse populations, is necessary to develop identification standards for forensic and physical applications. Furthermore, although the skull has been the focus of the majority of population affinity studies in forensic anthropology, other bones (e.g., femur and tibia) may also provide useful information [56]. Therefore, further studies investigating the feasibility of population affinity estimation based on other bones using CT images would be a useful addition to the extant literature that informs professional practice.

Most studies dedicated to population affinity estimation have analyzed the data obtained from physical specimens [13, 57, 58]. To the best of our knowledge, this study is the first to investigate the feasibility of population affinity estimation using 3D CT images of the pelvis. These images can reproduce complex curved features, and the data format facilitates statistically quantified computational geometrical analysis and the archiving case-related data [24, 59, 60]. Researchers have investigated the estimation of other biological attributes, such as sex, age, and stature, using pelvic CT images [29, 30, 61–63]. If CT data among institutions in different countries can be shared, it can facilitate the collection of multi-populational data and enable a deeper understanding of the diversity of pelvic morphology relative to morphometric population affinity variances.

It is important to that the present study is not without limitation. First, the data were collected from two different facilities using a 16-row and 64-row detector CT system, with different conditions for reconstructed images. Although these differences were unlikely to significantly affect the measurement data, it is more appropriate to use CT images from the same detector and under consistent conditions. Second, this study used PMCT and CT data of living patients. The shape and dimensions of human bones are not expected to change significantly after death; this study did not examine those differences. Third, the standard deviations for age in the Western Australian population were larger than those in the Japanese sample. Lovell [64] reported that the bone surfaces in older individuals are often highly irregular. Kolesova et al. [65] also reported that the pelvic size difference was associated with changes in age. Thus, including an elderly sample may have influenced the results of this study to some extent. Fourth, previous research has reported that the transverse diameter of the pelvic midplane and outlet can be influenced by hormone secretion at the end of pregnancy, leading to the softening of the pubic symphysis and pubic bone movement [66, 67]. However, in this study, information on whether female individuals had ever experienced pregnancy or childbirth was not available. Fifth, many of the measurements obtained in this study required an intact pelvis, and correct classification may not be possible if a fragmented pelvis is found. Finally, using morphometric geometric analysis could be able to detect other significant differences by detailing differences due to size and due to shape [7, 68, 69].

## Conclusions

The present study demonstrated that pelvic measurements derived in 3D CT images can be useful in the population affinity classification of Japanese and Western Australian individuals, especially in cases where the skull is unavailable in the forensic and anthropological contexts. Further research on the CT data of the pelvis involving other populations that have not been investigated is suggested. Moreover, further studies based on other skeletal measurements using CT images to estimate population affinity should be conducted.
